# Evolution of Swine Influenza Virus H3N2 in Vaccinated and Nonvaccinated Pigs after Previous Natural H1N1 Infection

**DOI:** 10.3390/v14092008

**Published:** 2022-09-10

**Authors:** Álvaro López-Valiñas, Laura Baioni, Lorena Córdoba, Ayub Darji, Chiara Chiapponi, Joaquim Segalés, Llilianne Ganges, José I. Núñez

**Affiliations:** 1 IRTA, Programa de Sanitat Animal, Centre de Recerca en Sanitat Animal (CReSA), Campus de la Universitat Autònoma de Barcelona (UAB), Bellaterra, 08193 Barcelona, Spain; 2 Unitat Mixta d’Investigació IRTA-UAB en Sanitat Animal, Centre de Recerca en Sanitat Animal (CReSA), Campus de la Universitat Autònoma de Barcelona (UAB), Bellaterra, 08193 Barcelona, Spain; 3 WOAH Collaborating Centre for the Research and Control of Emerging and Re-Emerging Swine Diseases in Europe (IRTA-CReSA), 08193 Barcelona, Spain; 4 WOAH Reference Laboratory for Swine Influenza, Istituto Zooprofilattico Sperimentale della Lombardia ed Emilia-Romagna, 25124 Brescia, Italy; 5 Departament de Sanitat i Anatomia Animals, Universitat Autònoma de Barcelona, Bellaterra, 08193 Barcelona, Spain; 6 WOAH Reference Laboratory for Classical Swine Fever, IRTA-CReSA, 08193 Barcelona, Spain

**Keywords:** swine influenza virus, viral evolution, next-generation sequencing (NGS), vaccine, hemagglutinin (HA), neuraminidase (NA)

## Abstract

Swine influenza viruses (SIV) produce a highly contagious and worldwide distributed disease that can cause important economic losses to the pig industry. Currently, this virus is endemic in farms and, although used limitedly, trivalent vaccine application is the most extended strategy to control SIV. The presence of pre-existing immunity against SIV may modulate the evolutionary dynamic of this virus. To better understand these dynamics, the viral variants generated in vaccinated and nonvaccinated H3N2 challenged pigs after recovery from a natural A(H1N1) pdm09 infection were determined and analyzed. In total, seventeen whole SIV genomes were determined, 6 from vaccinated, and 10 from nonvaccinated animals and their inoculum, by NGS. Herein, 214 de novo substitutions were found along all SIV segments, 44 of them being nonsynonymous ones with an allele frequency greater than 5%. Nonsynonymous substitutions were not found in NP; meanwhile, many of these were allocated in PB2, PB1, and NS1 proteins. Regarding HA and NA proteins, higher nucleotide diversity, proportionally more nonsynonymous substitutions with an allele frequency greater than 5%, and different domain allocations of mutants, were observed in vaccinated animals, indicating different evolutionary dynamics. This study highlights the rapid adaptability of SIV in different environments.

## 1. Introduction

Swine influenza viruses (SIVs) are the etiological agents of a pig respiratory disease that causes important economic loses in the pig industry. Although the mortality rate associated with SIV infection is usually low, it is a highly contagious disease with morbidities that can reach almost 100% of exposed animals [1,2]. SIVs belong to the *Orthomyxoviridae* family type A. The SIV genome, with approximately 13.6 kb size, is characterized by having 8 RNA negative polarity strain segments: the polymerases (polymerase basic 2 and 1 (PB2 and PB1) and acidic polymerase (PA)), the hemagglutinin (HA), nucleoprotein (NP), neuraminidase (NA), matrix (M), and the nonstructural (NS) [3].

The surface glycoproteins HA and NA are responsible for interacting with host cells through sialic acid, allowing the entry of viral particles and the exit of virions after the influenza A virus (IAV) infection cycle [4,5,6]. Both proteins generate different antigenic patterns used to classify IAVs. Nowadays, H1N1, H1N2, and H3N2 are the most prevalent circulating enzootic subtypes in the swine population worldwide [7]. Moreover, those proteins play an important role in generating protective immunity against SIV, more specifically against HA protein [8]. Currently, though its utilization is still limited, the trivalent vaccine, which contains the three previously mentioned SIV inactivated subtypes, is the most widely used to prevent and control the disease [8,9,10]. Although the vaccine can reduce disease and virus spread, the host immune system does not generate sterilizing immunity, so viral replication can occur despite vaccination [11,12].

Influenza viruses have a high evolutionary capacity to evade pre-existing immunity [13,14]. This ability is related to the low proof-reading fidelity of IAVs’ polymerase, which can lead to the accumulation of point mutations in the IAV RNA segments, achieving new antigenic patterns in a short time, a phenomenon known as antigenic drift [15,16]. HA and NA have a great relevance in the humoral response against IAVs. Punctual nonsynonymous substitutions in both the HA globular and NA head domains can interfere with host capacity to generate an effective antibody response to the virus [17,18,19]. Previous studies have shown that the lack of effectiveness of pre-existing immunity against IAVs may favor the virus’s evolution due to a strong antigenic selection driving the rapid generation of new epitopes [20,21].

During the 1990s, SIV H3N2 subtype arose from the triple-reassortment of HA, NA, and PB1 segments from seasonal human H3N2 and PB2, and PA from avian IAV, and NP, M, and NS from the classical H1N1 SIV [22]. This subtype was first identified in swine, and it has been circulating since then all over the world [22,23,24]. In 2009, A(H1N1) pdm09 triple-reassortment virus arose from the Eurasian “avian-like” swine H1N1 and swine triple-reassortments H1N2 and H3N2 [25]. Thereby, pigs play an important role in the IAVs’ adaptation to different hosts, avian to human and vice versa [26]. The current dominating IAV strains in the European swine population are “avian-like” swine H1N1, “human-like” reassortment swine H1N2, “human-like” reassortment swine H3N2, and A(H1N1) pdm09 virus [2].

Considering the enormous genetic variability and evolutionary capacity of IAVs, their endemic character, the continuous increase in the pig population around the world and the close contact among pigs, birds, and humans, IAV poses a constant threat to human and animal health. Thus, SIV evolution studies including determination of new viral variants emergency are fundamental to improve surveillance and disease control.

Against this background, the aim of the present study was to determine the evolutionary trends of SIV H3N2 subtype in vaccinated and nonvaccinated pigs in previously naturally H1N1 infected herds. To this end, 14 whole SIV genome samples were sequenced by next generation sequencing, and new viral variants were determined.

## 2. Materials and Methods

### 2.1. Cell and Viruses

Madin-Darby Canine Kidney (MDCK, ATCC CCL-34) cells were used for both viral titration and production. Dulbecco’s Modified Eagle Medium (DMEM), supplemented with fetal bovine serum (FBS) (10%), L-glutamine (1%) and penicillin/streptomycin (1%), was used for cell culture kept at 37 °C with 5% CO_2_ atmosphere in an incubator.

The A/Swine/Spain/SF32071/2007 H3N2 virus was propagated via infection of a MDCK monolayer cell culture, with addition of 10 µg/mL of porcine trypsin (Sigma-Aldrich, Madrid, Spain), at a multiplicity of infection (MOI) of 0.01. Forty-eight hours later, virus was harvested, and titration was performed by serial dilutions in a MDCK cell culture to calculate the 50% tissue culture infection dose (TCID_50_) according to the Reed and Muench method [27].

### 2.2. Experimental Design

Sixteen 5-week-old domestic pigs were selected from a Spanish commercial farm where the flu vaccine was not applied. Animals were distributed into two groups, Group A (animals from 1 to 8) and Group B (animals 9 to 16). After one week of acclimation period, animals from Group A were immunized with the first dose of the commercial inactivated influenza vaccine (RESPIPORC FLU3, IDT^®^, Dessau-Rosslau, Germany), according to the manufacturer’s instructions. In the vaccine formulation, the following strains are included H3N2 (Bakum/IDT1769/2003), H1N1 (Haselünne/IDT2617/2003), and H1N2 (Bakum/1832/2000). The vaccine was administered by the intramuscular route using a 2 mL dose injected in the neck muscle. Twenty-one days post-vaccination (dpv), Group A received the second vaccine dose, using the same protocol. In parallel, pigs from Group B were intramuscularly inoculated at 0 and 21 dpv with 2 mL of phosphate buffered saline (PBS).

Three weeks after the second vaccination dose (42 dpv), pigs from both experimental groups were challenged with A/Swine/Spain/SF32071/2007 (H3N2) by two administration routes, intranasal and endotracheal, with a viral concentration of 10^7^ TCID_50_ in a final volume of 2 and 5 mL per route, respectively [28]. For the viral challenge, animals were restrained with a pig lasso, and a diffuser device (MAD Nasal, Teleflex, Morrisville, NC, USA) was used to administrate intranasally 1 mL of inoculum per nostril; animals were intubated for the endotracheal administration [29].

Clinical signs, including rectal temperature and animal behavior, were daily measured by a trained veterinarian after challenge. The registration of clinical signs was scored as previously described [30]. The same number of animals per group was serially euthanized, three animals each at 2 and 5 days post-inoculation (dpi), and the remaining two at 9 dpi.

Nasal swabs and sera were collected before vaccination, viral challenge, and on day of euthanasia. In addition, nasal swabs were daily collected from 1 dpi until the euthanasia day. Lung, nasal turbinate (NT) and broncho-alveolar lavage fluid (BALF) were collected and stored at −80 °C. The BALF was collected by filling the right lung of each pig with 150 mL of PBS. An extra lung tissue was collected and fixed by immersion in 10% buffered formalin.

Animal experiments were performed in AM Animalia facilities (La Vall de Bianya, Girona, Spain). All procedures were approved by the animal ethics committee from the Generalitat de Catalunya, under project number 10442, following the Spanish and European regulations.

### 2.3. Humoral Response against SIV

The detection of antibodies against influenza NP was performed using the ID Screen^®^ Influenza A Antibody Competition ELISA kit (ID VET, Grabels, France). The inhibition percentage value was calculated, with values higher than 50% considered negative, those below 45% positive, and those between both values doubtful, according to manufacturer’s instructions.

Hemagglutination inhibition (HI) assay was performed at 42 dpv (challenge) and on euthanasia day as previously described [29]. Briefly, collected sera from pigs were treated 18 h at 37 °C with RDE II Seiken (Denka Chemicals, Tokyo, Japan) and inactivated for one hour at 56 °C. A volume of 50% chicken red blood cells (RBCs) was added to remove unspecific inhibitors by hemadsorption and subsequently diluted in PBS (1:10). Then, pig sera were twofold diluted in PBS until 1:1024 in v-bottomed 96-well plates. Twenty-five microliters of viral antigen was used on challenge (A/Swine/Spain/SF32071/2007 (H3N2)), diluted to 4 hemagglutination units (HAU) was dispensed. After 1 h of incubation at room temperature, 25 µL of 0.5% of chicken RBC was also added. Finally, after another hour of incubation, hemagglutination was evaluated by tilting the plate. The antibody titers were established as the reciprocal dilution where inhibition was complete, considering seroprotective titers ≥1/40.

### 2.4. Pathological Analyses in Lung and Immunohistochemistry to Detect SIV

Macroscopic examination of the lung parenchyma was performed during the necropsies of animals. Moreover, an image analysis was performed to calculate the percentage of lung-affected area, as previously described [31], with ImageJ^®^ software (v 1.8) [32].

A sample of each collected lung was fixed in 10% buffered formalin, dehydrated and embedded in paraffin wax. Two sections of 3–5 μm thick were cut and stained with hematoxylin-eosin (HE) for the examination under light microscopy [28,33] to detect SIV antigen by immunohistochemistry (IHC). For the latter, a two-step polymer method (Envision TM) was performed using a monoclonal antibody against influenza A virus (IAV) (Hb65 from the ATCC) and system + HRP-labeled polymer anti-mouse (K4001, Dako, Santa Clara, CA, USA) as primary and secondary antibodies, respectively [34]. To determine the degree of lung lesions, a semiquantitative method was used based on affected airways on HE examination; the amount of immunoreactivity on IHC examination was also assessed [35].

### 2.5. SIV RNA Detection

Lung and NT were homogenized in brain heart infusion medium (10% weight/volume). RNA was extracted from nasal swab, BALF, lung, and NT using the MagAttract 96 Cador Pathogen kit ^®^ (Qiagen, Düsseldorf, Germany) following the manufacturer’s instructions. Subsequently, the SIV RNA of each sample was quantified by RT-qPCR assay based on the amplification of the conserved segment of the matrix (M) gene [36]. The amplification was performed in the Fast7500 equipment (Applied Biosystem, Foster City, CA, USA). Samples where fluorescence was not detected were considered negative whereas threshold cycle (Ct) values under 40 were considered positive [12,36].

### 2.6. Whole Influenza Genome Sequence by NGS

The RNA extracted from samples of vaccinated and nonvaccinated animals with a RT-qPCR Ct value lower than 37 were used for whole genome amplification [12,37]. The PCR conditions were: 0.2 μM of forward and reverse primers MBTuni-12 and MBTuni-13, respectively, 2.5 μL of extracted RNA and 0.5 μL of SuperScript^®^ III One-Step RT-PCR System with Platinum™ Taq High Fidelity DNA Polymerase (Thermo Fisher Scientific, Waltham, MA, USA). Moreover, for the biggest segments (PB2, PB1 and PA), amplification was enhanced by a second amplification with the same conditions but adding a modification of forward primer (MBTuni12G) [38].

Those samples with the eight segments amplified were selected for sequencing [37] by Illumina technology. The Nextera-XT DNA Library Prep protocol (Illumina^®^, San Diego, CA, USA) was followed for multiplexed sequencing libraries and sequencing was performed by Miseq Reagment Kit v2 in a 150-cycle paired-end run, on a Miseq Instrument (Illumina^®^, San Diego, CA, USA).

Sequencing data were deposited at the National Center for Biotechnology Information (NCBI, https://www.ncbi.nlm.nih.gov/ (accessed on 10 May 2022)) with the accession number (PRJNA853173).

### 2.7. De Novo Assambly, Mapping, and Variant Calling

After the Miseq run, adapters were automatically removed and reads quality were analyzed by FastQC (v 0.11.8) [39]. All reads with a low quality (Phread < 30) were trimmed using Trimmomatic (v0.39) [40]. De novo assembly was performed using the SPAdes algorithm with the very sensitive option [41]. The assemblies generated were screened using NCBI BLAST (https://blast.ncbi.nlm.nih.gov/Blast.cgi (accessed on 10 May 2022)) to determine the strain. To obtain inoculum consensus sequence, inoculum sequencing reads were mapped against A/Swine/Spain/SF32071/2007 (H3N2) reference SIV genome sequences (HE774666-73) [42], using the very sensitive function of the read alignment tool Bowtie2 (v2.3.5) [43]. The consensus sequence was generated using the consensus option from Bcftools (v.1.9) [44]. To detect single nucleotide polymorphisms, reads obtained from each sequenced sample were mapped against it. Post-mapping, all reads with a mapping quality lower than 30 were removed from the analysis using Samtools (v.0.39) [44]. The Picard “MArkDuplicatesSpark” option and the “BaseRecalibrator” options, both included in GATK4 (v4.1), were used for removing PCR duplicates and reads recalibration, respectively. For each sample, the number of mapped reads against SIV whole genome and its depth were calculated with the “-depth” function included in Samtools (v1.9) and represented with RStudio using ggplot2 library [45,46].

All variants found in each sample were noted using the default parameters of LoFreq software [47]. A single nucleotide variant was considered if at least 50 reads of depth, 10 reads of the alternative base count, and *p* value < 0.01 requirements were obtained. The effect of each variant was predicted with SnpEff software (v.4.3) [48]. A previous database from the H3N2 subtype was built with “build–gtf22” function, using the previous annotated genome [42].

### 2.8. Nucleotide Diversity (π)

The genetic diversity of all sequenced samples per genomic segment was calculated with SNPGenie software (v 1.2) [49]. The nucleotide diversity (π) was calculated as the mean number of pairwise difference per site across each genomic segment. Moreover, the nonsynonymous and synonymous nucleotide diversity (π_N_ and π_S_) was calculated as the mean number of nonsynonymous and synonymous differences per nonsynonymous and synonymous sites in each genomic segment, respectively.

### 2.9. Lolliplot and 3D Structural Representation of SIV Proteins

The trackViewer package from Bioconductor was used to visualize the lolliplot proteins representation of PB2, PB1, PA, HA, NA, matrix (M1), and nonstructural 1 (NS1) [50]. Moreover, the PyMOL Molecular Graphics System Version 4.6 was used to perform the structural representation of HA and NA proteins. Indeed, all nonsynonymous variants with an allele frequency greater than 5% were pointed in all protein representations.

### 2.10. Satatistical Analysis

Statistical differences in rectal temperatures per day between both groups were studied by T-test application. Pearson’s chi-squared and Kruskal–Wallis tests were also applied to study significant differences between groups in the proportion of synonymous and nonsynonymous substitutions and nucleotide diversity, respectively.

## 3. Results

### 3.1. Detection of Natural SIV Circulation in Both Experimental Groups at Vaccination Day

**Figure 1:**
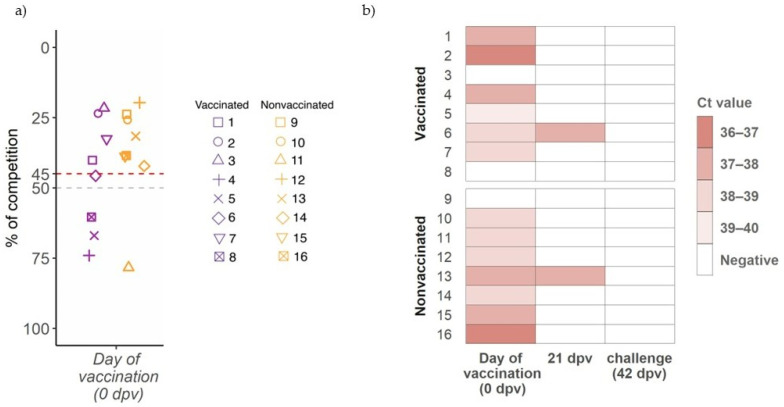
Detection of antibodies against influenza NP at day of first vaccination and SIV detection before challenge. (**a**) IgG antibody levels against NP detected by ELISA, where levels are shown on ordinate axis expressed as percentage of competition. Samples with a value greater than 45 (red line) are considered positive, below 50 (gray line) negative, and between both doubtful. Violet and orange boxplots and dots indicate samples from vaccinated and nonvaccinated groups. Each animal is represented by one sot shape. (**b**) SIV detection by RT-qPCR heatmap. Animal IDs are indicated in ordinate axis.

At first vaccination day (0 dpv), an antibody response against NP was already detected by ELISA test in pigs from both experimental groups (7 in vaccinated and 4 in nonvaccinated groups) (Figure 1a). Due to such unexpected antibody detection, SIV RNA detection was attempted and confirmed by RT-qPCR in 6 and 7 nasal swab samples from vaccinated and nonvaccinated animals, respectively. High Ct values between 36.3 and 39.14 were found (Figure 1b). Three weeks later (21 dpv), all animals were RNA SIV negative except one animal from each experimental group, being all animals RT-qPCR negative on the day of challenge (42 dpv) (Figure 1b).

### 3.2. Identification of H1N1 Subtype as Natural Circulating Virus before Challenge

After detection of the unexpected SIV infection, the characterization of the circulating virus was performed by NGS. The NCBI BLAST screening of de novo assembly indicated that the circulating strain detected was H1N1, more specifically a strain derived from A(H1N1) pdm09.

### 3.3. Humoral Response againts SIV after Vaccination and H3N2 Challenge

**Figure 2:**
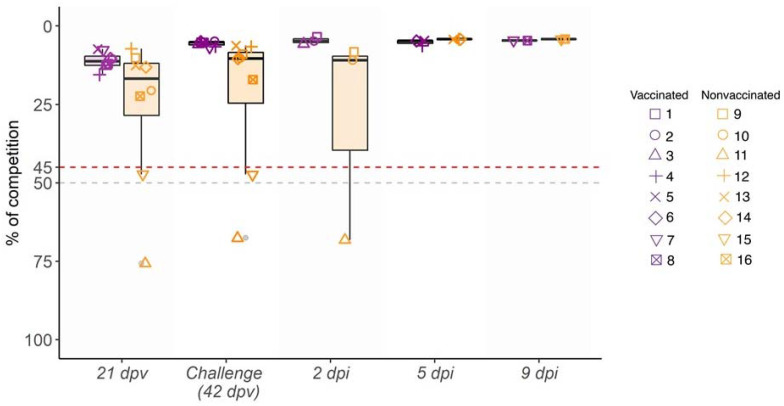
Kinetics of antibody levels against SIV NP in serum samples collected from 21 dpv to 9 dpi. Percentage of competition (%) and the day of sampling are represented in ordinate and abscissa axes, respectively. Violet indicates vaccinated animals, whereas the nonvaccinated group is indicated in orange box plots, where whiskers indicate quartiles’ variability. Each animal ID is represented by different shapes. Values plotted as the upper red line (<45%) are considered positive, whereas values of the lower gray line (>50%) are considered negative. Values between both lines (45% and 50%) are considered doubtful.

At three weeks after the first immunization (21 dpv) as well as on day of challenge (42 dpv), high levels of antibodies against NP were developed in all vaccinated animals, reaching values of 13.97% of competition detected by ELISA test. After the H3N2 SIV challenge, high antibody titers were detected, being similar at all time points until the end of the study (Figure 2). Meanwhile, 6 out 8 nonvaccinated animals were NP antibody positive with lower antibody values and heterogeneous response at 21 and 42 dpv. After challenge, from 5 dpi until the end of the study, the levels of antibodies from the nonvaccinated group reached the same values as those of vaccinated ones (Figure 2).

HI titers against H3N2 subtype were detected in all vaccinated pigs at 42 dpv, showing values greater than 40 in 5 out of 8 sera. After challenge, titers rapidly increased from 2 dpi to the end of the trial in all vaccinated animals with values from 40 to 320. By contrast, in nonvaccinated animals, HI titers were not detected until 5 dpi, with low values. However, at 9 dpi titers increased (Table 1).

### 3.4. Kinetics of Rectal Temperature after SIV Challenge

**Figure 3:**
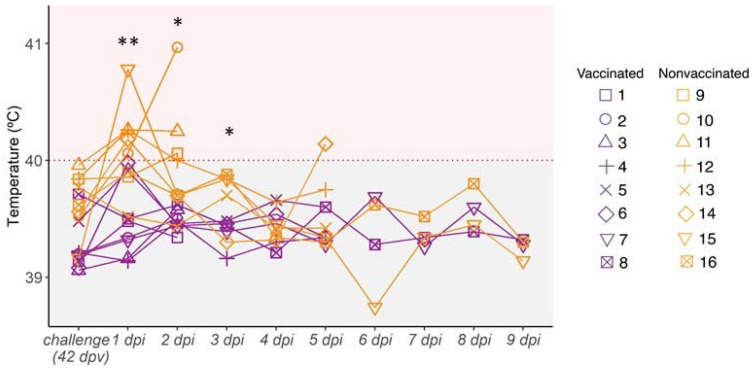
Kinetics of rectal temperature after H3N2 SIV challenge. Pigs are considered to have fever with temperatures greater than 40 °C (red dashed line). Vaccinated animals are represented in violet whereas nonvaccinated ones are represented in orange. Each line represents the rectal temperature measured in each pig over time; (*) *p* ≤ 0.05 and (**) *p* ≤ 0.01.

After H3N2 SIV challenge, most nonvaccinated animals had fever at 1 and 2 dpi. Afterwards, all animals displayed normal rectal temperature until the end of the study, except pig 14, which showed a fever peak at 5 dpi (Figure 3). By contrast, none of the vaccinated pigs developed fever during the trial (Figure 3). The rectal temperature recorded in nonvaccinated animals was statistically significant with respect to vaccinated ones at 1, 2, and 3 dpi (t-test; *p* = 0.002833, *p* = 0.02164, *p* = 0.03623, respectively).

### 3.5. SIV Genome Detection after Challenge

All vaccinated pigs were negative for SIV RNA detection in nasal swabs at 2 dpi. Later, only animal 4 tested positive at 3, 4, and 5 dpi (time of euthanasia). Meanwhile, pigs 5, 7, and 8 were positive at 5, 3, and 9 dpi, respectively. By contrast, all nonvaccinated pigs tested positive from 2 to 4 dpi, most showing Ct values below 33 (Table 2). From 5 to 9 dpi, all nasal swabs analyzed were RT-qPCR negative, except for those of animals 13 and 16 at 5 and 6 dpi, respectively.

In the case of BALF samples, SIV genome was detected in those collected from 3 vaccinated pigs at 2, 5, and 9 dpi. Likewise, RT-qPCR positive BALF samples were found in 4 nonvaccinated pigs, three at 2 dpi and the other at 5 dpi. In relation to lung samples, SIV was found only in the three vaccinated animals euthanized at 2 dpi, whereas all nonvaccinated animals were positive in all collected samples in the trial. Finally, all nasal turbinate samples from vaccinated animals were negative, while two samples from the nonvaccinated group were positive (Table 2).

From all the RT-qPCR positive samples, 16 SIV whole genomes were amplified and sequenced. From vaccinated animals, complete SIV sequences were obtained from 3 nasal swab samples (pig 5 from 3 to 5 dpi), two lung samples (pigs 2 and 3), and one BALF sample (pig 1). From nonvaccinated pigs, complete SIV genomes were obtained from seven nasal swab samples (pigs 9, 10, and 11 at 2 dpi, pigs 12, 13, and 15 at 3 dpi and pig 16 at 4 dpi), two lung samples (pigs 9 and 10) and one nasal turbinate sample (pig 10) (Table 2).

### 3.6. Lung Gross Lesions, Histopathological Assessment, and Immunohistochemistry to Detect SIV Antigen

On average, more extensive gross lung lesions were detected in nonvaccinated animals compared to vaccinated ones (Table 3). The highest percentages of lung affected area were recorded in animals 9 and 10 from the nonvaccinated group euthanized at 2 dpi, reaching values of 21.4%.

Microscopic lesions were detected in both groups, with a lower scoring in vaccinated animals at all sampling days. Remarkably, the highest scoring values were detected in all lung sections from nonvaccinated animals at 2 dpi, still being high at 5 dpi. No viral labeling by immunohistochemistry in lung sections was detected in vaccinated animals. In contrast, low to moderate immunoreactivity was found in three nonvaccinated animals (Table 3).

### 3.7. Whole SIV Genome Sequences Obtained from Vaccinated and Nonvaccinated Challenged Animals

**Figure 4:**
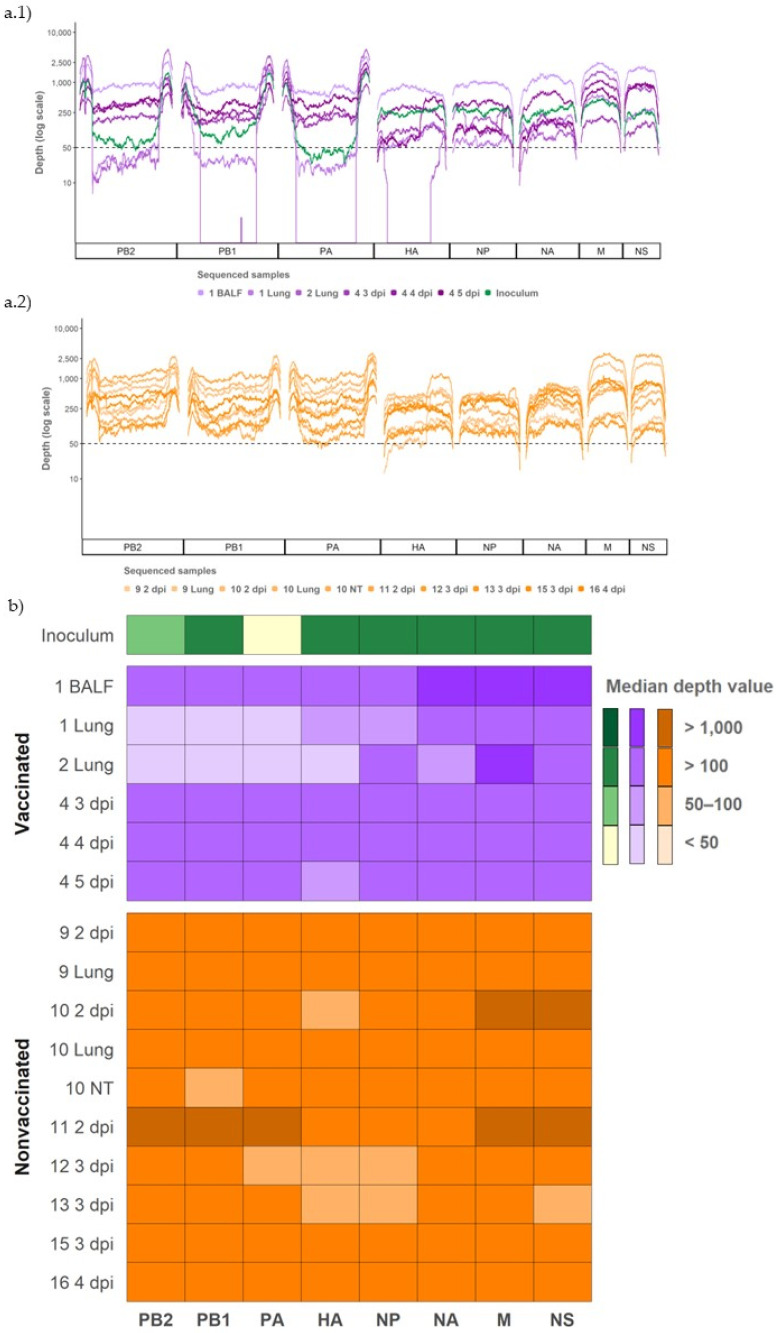
Coverage and depth obtained per genomic segment of SIV H3N2 in inoculum and vaccinated and nonvaccinated pig samples. (**a.1**) Illumina sequencing profile reads coverage against H3N2 SIV and its depth in inoculum (plotted in green) and samples from vaccinated animals (plotted in different tones of violet). (**a.2**) Illumina profile of samples collected from nonvaccinated animals plotted in different orange tones. (**b**) Heat map of the median depth value per genomic segment in all sequenced samples. The origin and the collection day of each sample are specified in the figure. Virus samples from BALF, lung, and NT were collected on indicated euthanasia day.

The complete inoculum sequence (A/Swine/Spain/SF32071/2007 H3N2 strain) used in the viral challenge was determined (Figure 4). In addition, 16 SIV whole genomes were retrieved from samples collected from challenged pigs (Table 1 and Figure 4). Notably, six sequences were recovered from vaccinated pigs and 10 from nonvaccinated ones. From all sequenced samples, a total of 1,198,942 reads were obtained after quality control and 84.36% of them (1,011,412) matched the SIV genome. The maximum number of mapped reads was obtained in the nasal swab collected from nonvaccinated animal 11 at 2 dpi (135,062). By contrast, the minimum number of reads was obtained in the nasal swab of the nonvaccinated animal 12 at 3 dpi (20,581) (Table S1).

Regarding coverage per position, 93.91% from all determined SIV genomes was represented with more than 50 reads and selected for further analysis (Figure 4(a.1,a.2)). Specifically, most genome segments were obtained from samples of vaccinated and nonvaccinated pigs, with a depth median greater than 50. Only the segments PB2, PB1, and PA from lung samples collected from vaccinated pigs 1 and 2, and the HA from lung collected also from vaccinated pig 2, had a depth median lower than 50 (Figure 4b).

### 3.8. Evolution of Variants Found in Inoculum

From the viral inoculum, five nonsynonymous variants were detected, all of them in the polymerase segments: D55N (2.98% allele frequency), E617A (19.05%), V618I (17.5%) and L683I (5.09%) in PA, and Q73K (2.32%) in PB2 (Table 4).

**Figure 5:**
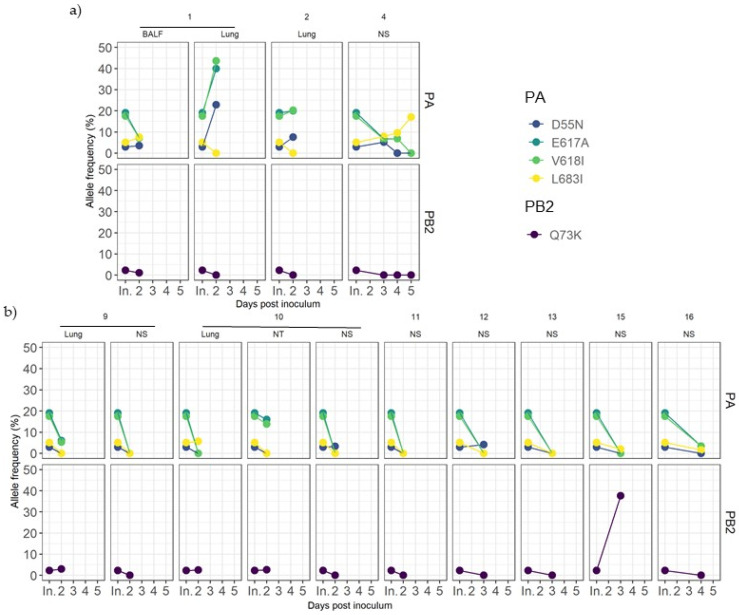
Dynamics of inoculum variant frequencies over time. (**a**) Samples from vaccinated animals. (**b**) Samples from nonvaccinated animals. Rows indicate genomic segments PA and PB2. Columns correspond to each animal and sample origin. X axes correspond to post-inoculation time in which each sample was recovered, where “In.” indicates the inoculum. Y axes show the allele frequency. Each substitution is plotted in a different color.

Those variants were later detected in sequenced samples from both experimental groups (Figure 5). For most samples collected during the trial, these substitutions were maintained at the same frequency, or they were lost over time, with a few exceptions. In vaccinated pigs, PA substitutions D55N, E617A, and V618I showed a rapid allele frequency increase in the lung sample from animal 1. The allele frequency of detection of the L683I substitution in PA also increased over time in nasal swabs collected from pig 4 (Figure 5a). On the other hand, in nonvaccinated animals, PB2 Q73K substitution considerably increased its frequency in the nasal swab sample from animal 15 at 3 dpi (Figure 5b).

### 3.9. Detection of De Novo SIV Variants in Sequenced Samples Collected from Vaccinated and Nonvaccinated Animals

**Figure 6:**
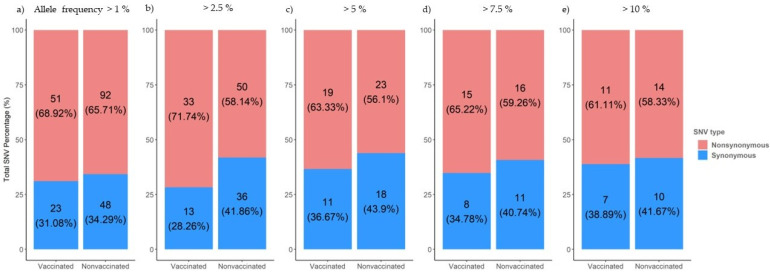
Proportion of synonymous and nonsynonymous de novo SNVs generated in sequences collected from vaccinated and nonvaccinated pigs. Substitutions with an allele frequency greater than 1% (**a**), 2.5% (**b**), 5%(**c**), 7.5% (**d**), and 10% (**e**) are represented.

From samples collected during the trial, single nucleotide variants (SNV) were noted for an allele frequency of 1, 2.5, 5, 7.5, and 10% (Figure 6). The proportion of nonsynonymous variants was higher than that of the synonymous ones for the 5 allele frequencies analyzed (Figure 6). No significant differences between groups were found applying Pearson’s chi-squared test.

**Figure 7:**
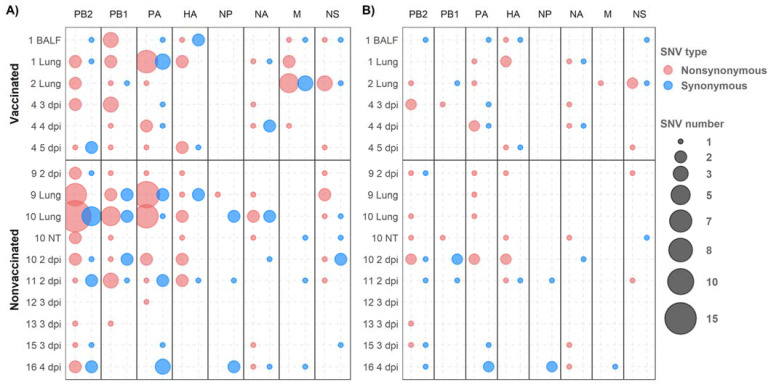
De novo synonymous and nonsynonymous SNV allocation. Nonsynonymous and synonymous variants are represented in red and blue, respectively. Circle size indicates the total number of substitutions found per genomic segment (abscissa axis) and sample (ordinate axis). (**A**) SNVs with an allele frequency greater than 1%. (**B**) SNVs with an allele frequency greater than 5%.

The synonymous and nonsynonymous variants were allocated in all genomic segments recovered from both experimental groups (Figure 6). Regarding SNV with an allele frequency greater than 1%, 56, 33, and 49 were detected in polymerase segments PB2, PB1, and PA, respectively. Notably, most of them were nonsynonymous substitutions (Figure 7A). By contrast, the most of these variations did not exceed an allele frequency of 5% (Figure 7B); we detected only 16 in PB2, 6 in PB1, and 14 in PA(Figure 7B).

In relation to HA and NA segments, 20 and 16 SNVs were detected, respectively, of which nine and six were nonsynonymous variants with an allele frequency greater than 5% (Figure 7). In the NP gene, no nonsynonymous variants were found with an allele frequency greater than 5% (Figure 7B). Notably, in the M segment, 16 SNVs were detected with an allele frequency greater than 1%, nine of which were nonsynonymous substitutions found only in samples from vaccinated animals. Only one nonsynonymous and one synonymous substitution in the M segment gene were detected with an allele frequency greater than 5% (Figure 7). Lastly, in the NS segment, 19 SNVs were detected, with eight variants exceeding 5% of allele frequency, five of them being nonsynonymous ones (Figure 7).

**Figure 8:**
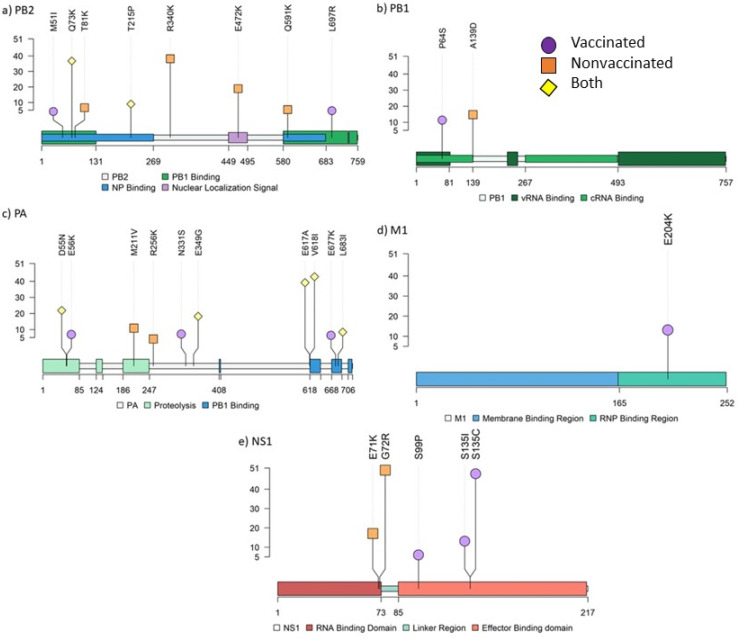
Lolliplot representing the allocation of all amino acid substitutions with an allele frequency greater than 5% found in PB2 (**a**), PB1 (**b**), PA (**c**), M1 (**d**), and NS1 (**e**) SIV proteins. Allele frequency of each variant is indicated on ordinate axis. If one substitution was found in more than one sample, only the highest allele frequency is indicated. Amino acid substitutions from vaccinated, nonvaccinated, and both groups are represented in purple circles, orange squares, and yellow diamonds, respectively.

**Figure 9:**
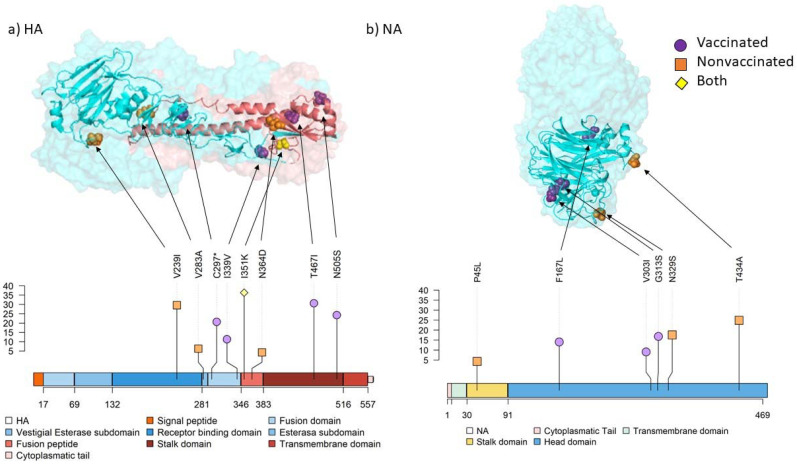
Location of substitutions with an allele frequency greater than 5% found in surface glycoproteins by both protein structure and lolliplot representation. The allele frequency is indicated on ordinate axis. (**a**) HA 3D trimer (PDB accession no. 7VDF) [51] and lolliplot domain representation. HA1 domains are represented in different tones of blues and HA2 domains in different tones of reds. (**b**) NA 3D tetramer head domain (PDB accession no. 4GZO) [52] and lolliplot domains representation. Substitution P45L from the stalk domain is not included in the limits of the crystalography structure used.

In vaccinated animals, 16 amino acid substitutions with an allele frequency greater than 5% were identified in PB2 (M51I and L697R), PB1 (P64S), PA (E56K, N331S, and E677K), HA (I339V, T467I, and N505S), NA (F167L, V303I, and G313S), M2 (E70K), and NS1 (S99P, S135I, and S135C). By contrast, 14 nonsynonymous substitutions were detected in nonvaccinated animals: PB2 (T81K, R340K, E472K, and Q591K), PB1(A139D), PA (M211V and R256K), HA (V239I, V283A, and N364D), NA (P45L, N329S, and T434A), and NS1 (G72R) (Figure 8; Figure 9). Further information about all substitutions with an allele frequency greater than 1% is noted in the Supplementary data (Table S3).

In samples sequenced in animal 4 at different time points, nonsynonymous substitutions PA L697R and NS1 S135I, and synonymous substitutions PA G1485A, HA A1500G, and NA A405G were detected in several samples. Indeed, in lung and NT samples collected in nonvaccinated animal 10, the nonsynonymous variant L655V was found (Table S2).

Nonsynonymous substitutions PB2 T2090G (L697R) and NS1 A403T (S135I), and synonymous substitutions PA G1485A, HA A1500G, and NA A405G were found in animal 4 at different time points. Indeed, nonsynonymous variant PA T1993G (L655V) was noted in lung and NT from animal 10 (Table S2).

Regarding stop-gained mutations, 11 different mutations were recorded only in lung samples from vaccinated animal 1 and nonvaccinated animals 9 and 10. Mutation S86* in PB2 was found in nonvaccinated animals 9 and 10, whereas E739* in PB1 was found in animals 1 and 10 (Table S3). Notably, all these substitutions were noted with an allele frequency lower than 5%, except C289* in HA from animal 1, which was represented with an allele frequency of 21.43% (Figure 9, Table S3).

### 3.10. Genetic Diversity in Viral Populations

**Figure 10:**
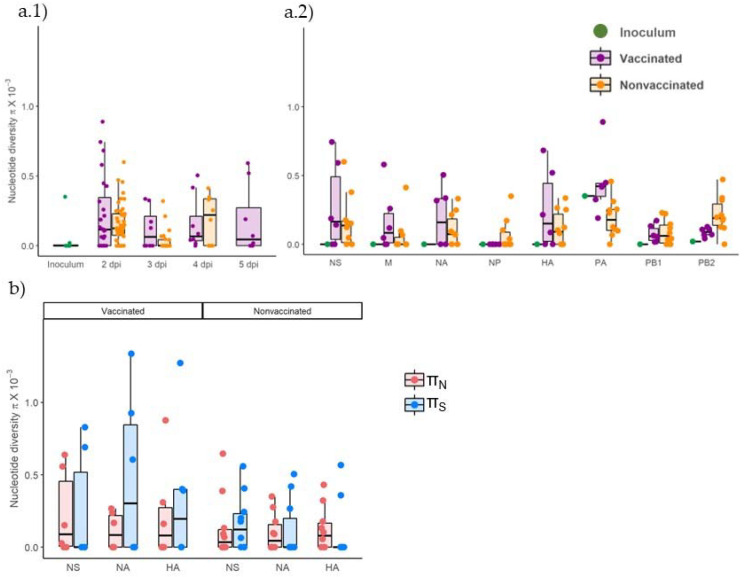
Genetic diversity in viral populations collected from vaccinated and nonvaccinated animals. (**a**) Nucleotide diversity (π) box plot representation of total viral population collected from inoculum, vaccinated, and nonvaccinated animals, plotted in green, violet, and orange, respectively. (**a.1**) Whole genome π per collection day (**a.2**) Total π per genomic segment. (**b**) Nonsynonymous and synonymous nucleotide diversity (πN and πS) box plot representation of all sequenced samples for NA, HA, and PA segments. The πN is represented in blue whereas πS is represented in red. Mean, lower, and upper quartiles are represented in each box plot, where whiskers indicate the variability outside quartiles. Points represent each analyzed sample.

Nucleotide diversity (π) increased in both experimental groups at similar levels at 2 dpi with respect to inoculum (Figure 10(a.1)). At 3 dpi, π decreased in viral samples from nonvaccinated animals and then increased at 4 dpi. In contrast, in vaccinated animals, π decreased slightly at 3 dpi and remained constant the rest of the days.

Regarding π by genomic segments, it increased in most samples in both experimental groups compared with the inoculum, in which only diversity was found in PA and PB2 segments (Figure 10(a.2), Table 4). The π only decreased in comparison with inoculum in the segment PA in virus collected from nonvaccinated animals.

According to the nonsynonymous and synonymous nucleotide diversity (πN and πS, respectively), on average, the πS was greater than πN in NA and HA segments in viral samples collected from vaccinated animals, whereas it was lower in NS. Meanwhile, in samples from nonvaccinated pigs, πS was greater than πN in the NS segment but lower in NA and HA segments (Figure 10b). No significant differences between groups were found applying the Kruskal–Wallis test.

## 4. Discussion

Swine influenza viruses are pathogens distributed worldwide in pigs that cause an important economic loss in the ever-growing pork production industry [7,53]. According to the Food and Agriculture Organization (FAO), Spain is the fourth country in the world in terms of the largest pig production, behind China, USA, and Brazil [53]. There are several SIV subtypes worldwide co-circulating in the field, namely H1N1, H3N2, and H1N2 [54,55], with high seroprevalence levels in farms, including in Spain [56]. Nowadays, together with biosecurity measures, vaccination, although limited, is one of the most used strategies to avoid the disease although it does not prevent virus replication [8,12,57]. The latter, together with the current increase in pig population and the capacity of SIV evolution, could change viral host range, antigenicity, and virulence. A previous study showed different evolutionary trends according to pig vaccination status, showing positive selection pressure in trivalent vaccinated animals, whereas purifying selection pressure was noticed in nonvaccinated ones [12]. Considering the capability of IAVs to escape pre-existing immunity acquired by natural infections and vaccine application [14], both scenarios were studied herein. Hence, for a better understanding of the mechanisms that shape the evolution trends of SIVs, a differential whole SIV genomic analysis was performed by deep sequencing after an experimental H3N2 inoculation in vaccinated and nonvaccinated pigs that had suffered a previous natural infection by A(H1N1)pdm09.

In the present study, antibodies against NP and SIV RNA were unexpectedly detected in pigs the day of the first vaccination (0 dpv). Viral and antibody detection methods evidenced that virtually all animals had a previous contact with an IAV before challenge. After genome detection and sequencing, the SIV A(H1N1)pdm09 genome was determined in one of these positive samples. Consequently, this virus was circulating in the farm, probably unnoticed, which highlights the high prevalence of this virus in the field, as previously reported [56]. Animals showed levels of antibodies against NP until the end of the experiment, these values being more heterogeneous in nonvaccinated animals. On the day of challenge, only sera from vaccinated pigs showed HI activity against the SIV H3N2 strain. Consequently, the viral replication was lower, and the pathological findings were milder in vaccinated animals, proving again the efficacy of the vaccine [12]. Furthermore, the clinical manifestation of the disease in nonvaccinated animals previously naturally infected with the A(H1N1)pdm09 pandemic virus does not provide apparent cross-protection against the H3N2 SIV subtype [58]. However, as the vaccine does not confer sterilizing protection, viral shedding and clinical–pathological features were also observed in vaccinated animals, as previously described [12,59]. The vaccine effect had a direct impact on sequencing results as viral shedding is lower in vaccinated animals, thus NGS coverage and depth were less favored in collected samples from vaccinated animals. Nonetheless, whole SIV genome sequences could be obtained from six samples recovered from vaccinated animals, allowing the possibility to compare the genetic variability in the viral population collected from both experimental groups. The percentage identity among challenge, vaccine, and pandemic strains HA and NA proteins are available in the Supplementary Table S4.

The virus used for pig inoculation was generated after three passages on MDCK cells. This virus, A/Swine/Spain/SF32071/2007 (H3N2), was isolated from a Spanish porcine influenza outbreak [42]. Regarding the genetic diversity found in the inoculum, only five nonsynonymous substitutions were found in PA and PB2 segments. According to the literature, PA D55N substitution was identified as a possible adaptation of IAV H3N2 subtype virus to mammals [60]. Furthermore, the substitution V618I was already noted in a previous similar study, where it was found at 5 dpi in one nonvaccinated animal challenged with H1N1 subtype [12]. In relation to the Q73K substitution found in PB2, it was already detected in the human influenza isolate A/Anhui/1/2013 (H7N9) [61]. Most of the substitutions found in the inoculum were later lost in the sequenced samples from animals after challenge; this result could indicate that these substitutions are implied in the adaptation of field virus in MDCK cells, and they are lost with the readaptation to the natural host. However, the allele frequencies of some substitutions tended to increase over time in some analyzed samples; thus, the presence of these substitutions does not seem to affect the virus fitness when it is readapted to pig.

Regarding de novo nonsynonymous and synonymous SNV proportions, they were greater in both experimental groups at all analyzed frequencies, although always slightly higher in samples of vaccinated pigs. Globally, the de novo analysis may support that under both scenarios (vaccination or not), the viral evolution of SIV H3N2 was under positive selection pressure, suggesting that virions are poorly adapted to swine hosts [62]. This positive evolutionary pressure could be influenced by the readaptation of virions from cell culture to their natural host or by host immune pressures.

From all sequenced samples collected from vaccinated and nonvaccinated animals, a total of 214 de novo SNVs were detected, proving once again the high mutation rate of RNA viruses such as IAVs [12,16,63]. The appearance of new variants with increased fitness could help virus adaptation to a novel host environment, those variants being naturally selected and therefore perpetuated in viral populations. However, many of these mutations may have a negative effect on the fitness of the virus. For instance, in the polymerase segments (PB2, PB1, and PA), 99 nonsynonymous variants were detected but only 21 exceed 5% of the allele frequency, showing that most mutations that are generated in these segments do not represent a benefit in viral fitness, probably affecting their replicative capacity.

In relation to the polymerase segments, in the PB2 protein responsible for host pre-mRNA cap binding [64], M51I and L697R, both retrieved in samples from vaccinated animals, were allocated in the PB1 binding domain of the protein [65]. Notably, substitution R340K was found in the nonvaccinated animal 13 with an allele frequency of 39.13%. According to previous studies, this substitution contributes increasing the activity of the polymerase, mammal adaptation, and virulence of H10N8, H7N9, and H9N2 avian IAV [66,67]. Therefore, this substitution may affect viral replication since it was found in the unique lung sample where immunoreactivity was detected at 5 dpi. The Q591K substitution was observed in the nonvaccinated animal 10, which showed one of the highest pathological scores and the highest rectal temperature registered in the study. This substitution was previously related to mammal adaptation and increased virulence of H5N1, H7M9, and H9N2 IAVs [68]. In the protein with polymerase activity, PB1, the substitution P64S was found in the vaccinated animal 4; this mutation has been previously found circulating in the swine population from Guangdong (China) in an SIV H1N1 virus [69]. With reference to the PA protein, whose key role is cleavage host pre-mRNA and transcription initiation [64], the substitution R256K noted in nonvaccinated animal 10 was found predominant in IAVs H1N1, H2N2, H3N2, and H5N1 [70]. Furthermore, the substitution E349G was found in samples collected from the vaccinated animal 4 and nonvaccinated animals 9 and 10. In previous works, this substitution has been shown to increase the virulence of IAV [71,72].

NS1 protein is implied in viral replication and host immunity regulation. In the present study, five nonsynonymous substitutions with an allele frequency greater than 5% were found in this protein: two in the RNA binding domain in samples from nonvaccinated animals and three in the effector binding domain from vaccinated ones. The two substitutions found in nonvaccinated animals, E71K and G72R, were located close to the linker region of the protein, which play an important role in its plasticity. Hence, these substitutions could be involved in different protein conformations, affecting their interaction with other molecules and therefore functions [73]. Moreover, the E71K substitution was previously studied by reverse genetics in avian IAV, and it reduced IFN-β expression [74]. This substitution was found in the viral population collected from the nasal swab of the nonvaccinated animal 10. Thus, the E71K substitution may limit IFN-β expression by increasing virus replication and virulence [75]. By contrast, in the vaccinated animal 2, substitutions S99P and S135I were simultaneously found. The first one interacts with the cellular polypeptide tripartite motif-containing protein 25 (Trim25), which regulates the host innate immune response to infection by ubiquitin ligases [76,77]. The second one is implied in host p85β binding, which interacts with phosphoinositide 3-kinase (PI3K), activating a cellular pathway resulting in inhibition of apoptosis by phosphorylation of caspases, contributing to virus replication and virulence [78,79]. Another substitution at position 135 (S135C) was found in the nasal swab from animal 4 at 4 and 5 dpi. It should be highlighted that allele frequency of this substitution increased from one day to the other from 6.35 up to 48.85%. Therefore, this substitution may benefit the viral fitness because of the rapid allelic increase and the viral shedding recorded during 3 consecutive days from this animal. In a previous study, nonsynonymous substitutions in this protein were only found in vaccinated animals, showing that this protein could rapidly change, facilitating the adaptability of the virus under immune pressure [12].

In relation to the M segment, nonsynonymous substitutions were only found in viral populations collected from vaccinated animals. From the seven substitutions identified, only M1 E204K found in the animal 2 exceeded 5% of allelic frequency (14.28%). The role of this protein is virion assembly by interaction with proteins and lipids [80]. A previous study revealed that at this position, it induces filamentous changes in infected cells, affecting virus growth [81]. Notably, only one nonsynonymous substitution was found in the NP protein with an allele frequency of 2.9%. This protein interacts with polymerases and has an important role in the synthesis of RNA and its traffic [82,83]. Nonsynonymous substitutions in this protein could impair viral fitness, as it is a highly conserved protein among IAVs [84]. Therefore, our findings could support NP as a good therapeutic target for antiviral and universal vaccine development [85,86,87]. However, in a previous study from our group, in which the evolutionary capacity of the H1N1 virus was evaluated, six nonsynonymous substitutions were described in this protein [12].

The surface glycoproteins HA and NA are the main targets to generate antibodies against IAV after natural infection and/or vaccination [20]. Therefore, substitutions in these proteins may play an important role in the generation of mutants that may allow viruses to escape pre-existing immunity. The πN and πS proportion was lower in vaccinated animals, whereas the opposite was found in nonvaccinated ones, pointing to different evolutionary patterns depending on immunological pressure. Thus, under both scenarios, these segments showed different evolutionary tendency, acting to purify and enable natural selection in viruses collected from vaccinated and nonvaccinated animals, respectively. Notably, on average the π was greater in vaccinated animals, and this could indicate that HA and NA are evolving faster in these animals, regardless of whether those substitutions were synonymous or not. Regarding substitutions found with an allele frequency greater than 5%, more changes were found, proportional to the number of obtained sequences per group, in samples from vaccinated animals.

In nonvaccinated animals, three substitutions were identified in the HA segment. Substitution V239I was the only one found in the receptor binding domain and it is allocated in one epitope region. This substitution was previously identified in epidemic H3N2 SIV subtype isolated in Southern China in 2012 [88]. Furthermore, V283A and N364D substitutions were found in the vestigial esterase domain and in the fusion peptide. In contrast, four substitutions were found in the fusion (C297* and I339V) and the stalk domains (T467I and N505S) in vaccinated animals. The stop codon mutation, originated by C297* mutation, generates a truncated protein with 296 amino acids. In a previous study, stop codon substitutions were found in HA and those substitutions were transmitted among pigs [89]. Furthermore, avian IAV HA truncated proteins have been previously found in environmental reservoirs [90]. The I339V substitution, close to the cleavage domain, has been previously reported in H3N2 canine IAV in China [91]. Regarding the stalk domain, substitutions T467I and N505S were simultaneously found in the lung sample of the vaccinated animal 2, with allele frequencies of 31.25 and 25%, respectively. According to a previous study, evolution of this domain is slower and independent from immune pressure [92]. Interestingly, our findings suggest that pre-existing immunity may have an implication in the positive evolution of substitutions in this domain, as no substitutions were found in the head domain. Finally, the substitution I351K in the fusion peptide was found in three samples from both experimental groups.

Regarding the NA segment, substitutions N329S and T434A, located in the head domain, were recovered from nonvaccinated animals 10 and 16, respectively. In a previous study, substitution N329S was associated with losing one glycosylation site, although it did not alter the activity of the protein [93]. According to a previous study, this substitution is allocated in the antigenic epitope F′329–339 [94]. In vaccinated animals, substitution F167L was recovered in animal 2, whereas V303I and G313S ones were detected in nasal swab samples from animal 4 at 3 and 4 dpi, respectively. Substitution V303I was previously reported circulating in Bulgaria during the 2019–2020 winter season in H3N2 human IAV [95]. This substitution is allocated in the epitope region E′302-308 of the N2 protein [94]. Considering that this substitution, like the previously mentioned S135C in NS1 protein, was found in nasal swab samples collected in the only vaccinated animal which showed SIV detection during three consecutive days, this substitution may favor at 3 dpi some adaptative advantage to viral replication.

In the present study, the high mutability rate of SIV has been further demonstrated [12], showing that IAVs’ genomes is constantly changing. The high population of pigs and the high persistence of the virus make IAVs a continuous threat to both animal and human health, as virus evolution could affect viral host range, antigenicity, and virulence. Hence, rapid virus evolution contributes to the fact that the virus remains in the field continuously, which simultaneously implies that the virus is constantly evolving. Herein, the impact of each de novo substitution found on virus fitness is hypothesized according to previous literature. Therefore, future research will be necessary to deeply understand the role of these substitutions, either alone or together, through reverse genetic technology studies [96]. Altogether, the risk of future pandemic IAVs, as happened in 2009, is not negligible. To avoid or minimize the damage it could cause, the reduction of SIV circulation as much as possible by applying stricter vaccination schedules and a more rational swine production are highly advisable. Furthermore, SIV surveillance studies in farms and more effective or universal vaccine development are also crucial to prevent future flu pandemics.

## Figures and Tables

**Table 1:** HI activity of sera against challenge strain A/Swine/Spain/SF32071/2007 (H3N2).

		HI Antibody Titer Against Challenge Strain in Sera			
Group	Pig ID	42 dpv	Euthanasia Day		
			2 dpi	5 dpi	9 dpi
Vaccinated animals	*1*	10	40		
	*2*	40	320		
	*3*	20	160		
	*4*	80		80	
	*5*	80		160	
	*6*	20		80	
	*7*	40			160
	*8*	40			80
	mean	41.3	173.3	106.7	120
Nonvaccinated animals	*9*	0	0		
	*10*	0	0		
	*11*	0	0		
	*12*	0		10	
	*13*	0		20	
	*14*	0		0	
	*15*	0			640
	*16*	0			320
	mean	0	0	10	480

**Table 2:** SIV detection in nasal swab samples collected at different time points and from BALF, lung, and nasal turbinate. RT-qPCR Ct values results are shown in the table. Neg.: negative. Highlighted cells represent sequenced samples. Violet and orange cells indicate the group of each animal, vaccinated and nonvaccinated, respectively.

Group	Pig ID	Nasal Swab Samples									Euthanized Day	Tissues Samples		
		1 dpi	2 dpi	3 dpi	4 dpi	5 dpi	6 dpi	7 dpi	8 dpi	9 dpi		LUNG	NT	BALF
Vaccinated animals	1	Neg.	Neg.								2 dpi	32.96	Neg.	34.45
	2	Neg.	Neg.									34.48	Neg.	Neg.
	3	Neg.	Neg.									39.36	Neg.	Neg.
	4	Neg.	Neg.	33.45	32.56	37					5 dpi	Neg.	Neg.	39.58
	5	Neg.	Neg.	Neg.	Neg.	37.42						Neg.	Neg.	Neg.
	6	Neg.	Neg.	Neg.	Neg.	Neg.						Neg.	Neg.	Neg.
	7	Neg.	Neg.	38,56	Neg.	Neg.	Neg.	Neg.	Neg.	Neg.	9 dpi	Neg.	Neg.	38.09
	8	Neg.	Neg.	Neg.	Neg.	Neg,	Neg,	Neg.	Neg.	37.23		Neg.	Neg.	Neg.
Nonvaccinated animals	9	Neg.	31.21								2 dpi	26.49	Neg.	35.98
	10	Neg.	33.42									25.27	31.68	34.88
	11	39.48	33.39									33.34	Neg.	34.14
	12	Neg.	38.28	30.98	Neg.	Neg.					5 dpi	34.41	Neg.	Neg.
	13	37.28	35	30.02	33.6	39.48						34.36	39.5	37.14
	14	Neg.	38.75	36.96	Neg.	Neg.						35.38	Neg.	Neg.
	15	Neg.	37.13	32.45	34.93	Neg.	Neg.	Neg.	Neg.	Neg.		39.78	Neg.	Neg.
	16	Neg.	Neg.	32.8	30.17	Neg.	34.64	Neg.	Neg.	Neg.	9 dpi	39.54	Neg.	Neg.

**Table 3:** Pathological results based on gross (percentage of lung-affected area) and histopathological observations, including the semi-quantitative scorings for HE and IHC in lung samples.

Group	Pig ID	Euthanasia Day	Lung Affected Area (%)	Histopathological Scoring	Immunohistochemistry for SIV
Vaccinated animals	1	2 dpi	1.44	2.5	-
	2		0.32	2	-
	3		0.32	2	-
	4	5 dpi	0.18	1.5	-
	5		0	0.5	-
	6		10	2.5	-
	7	9 dpi	1.25	0.5	-
	8		0	0	-
		mean	1.69	1.44	
Nonvaccinated animals	9	2 dpi	21.4	3	++
	10		20.38	3	+++
	11		1.54	3	-
	12	5 dpi	7.3	2.5	-
	13		2.07	2	+
	14		4.98	3	-
	15	9 dpi	1.11	1	-
	16		0.69	1.5	-
		mean	7.43	2.38	

Histopathology scoring: absence (0), few inflammatory cells isolated (0.5), localized cluster of inflammatory cells (1), several clusters of inflammatory cells (1.5–2), severely several (2.5), and many (3) airways affected. Moreover, minimal (1.5), mild (2) interstitial infiltrate, and plus moderate interstitial and alveolar infiltrates (2.5–3). SIV immunohistochemical scoring: absence (-), low (+), scattered (++), moderate (+++), and abundant (++++) amount of immunoreactivity.

**Table 4:** SNV detected in the inoculum.

	Gene	Depth of Read	Nucleotide Change		Alt. Base Count	Allele Frequency	Aminoacidic Change	
			Position	Ref. → Alt.			Ref. → Alt.	Position
Inoculum	PA	503	G → A	163	15	2.99	D → N	55
		147	A → C	1850	28	19.05	E → A	617
		160	G → A	1852	28	17.5	V → I	618
		1375	C → A	2047	70	5.09	L → I	683
	PB2	863	C → A	217	20	2.32	Q → K	73

Abbreviations: Alt. (alternative) and Ref. (reference).

## Data Availability

Sequencing data were deposited at NCBI, with the accession number (PRJNA853173).

## Supplementary Materials

The following are available online at https://www.mdpi.com/article/10.3390/v14092008/s1, Supplementary Table S1. Sequenced reads obtained per sample. The total number of reads and reads mapped against reference genome after post-mapping filter application. Supplementary Table S2. SNV and its allele frequency reported in several samples. Supplementary Table S3. SIV H3N2 single nucleotide variants detected in all samples collected from vaccinated and nonvaccinated pigs. Table S4. HA and NA amino acid percentage identity among challenge, vaccine, and pandemic strains.
